# Dermoscopy of discoid lupus in eyelids

**DOI:** 10.1016/j.jdcr.2026.08.018

**Published:** 2026-08-22

**Authors:** Ludwin F. Castro, Rebeca Aviles, Karla Osorio, Karla Figueroa, Amadeo Sandoval, Luz Ely Cerritos

**Affiliations:** Department of Dermatology, Hospital Nacional Zacamil, El Salvador

**Keywords:** dermoscopy, discoid lupus, eyelids

## Clinical presentation

A 26-year-old woman presented with erythematous atrophic plaques on the upper eyelids, periorbital regions, and nasal dorsum for 10 months (Fig 1). The diagnosis of discoid lupus erythematosus was confirmed histopathologically.

**Fig 1:**
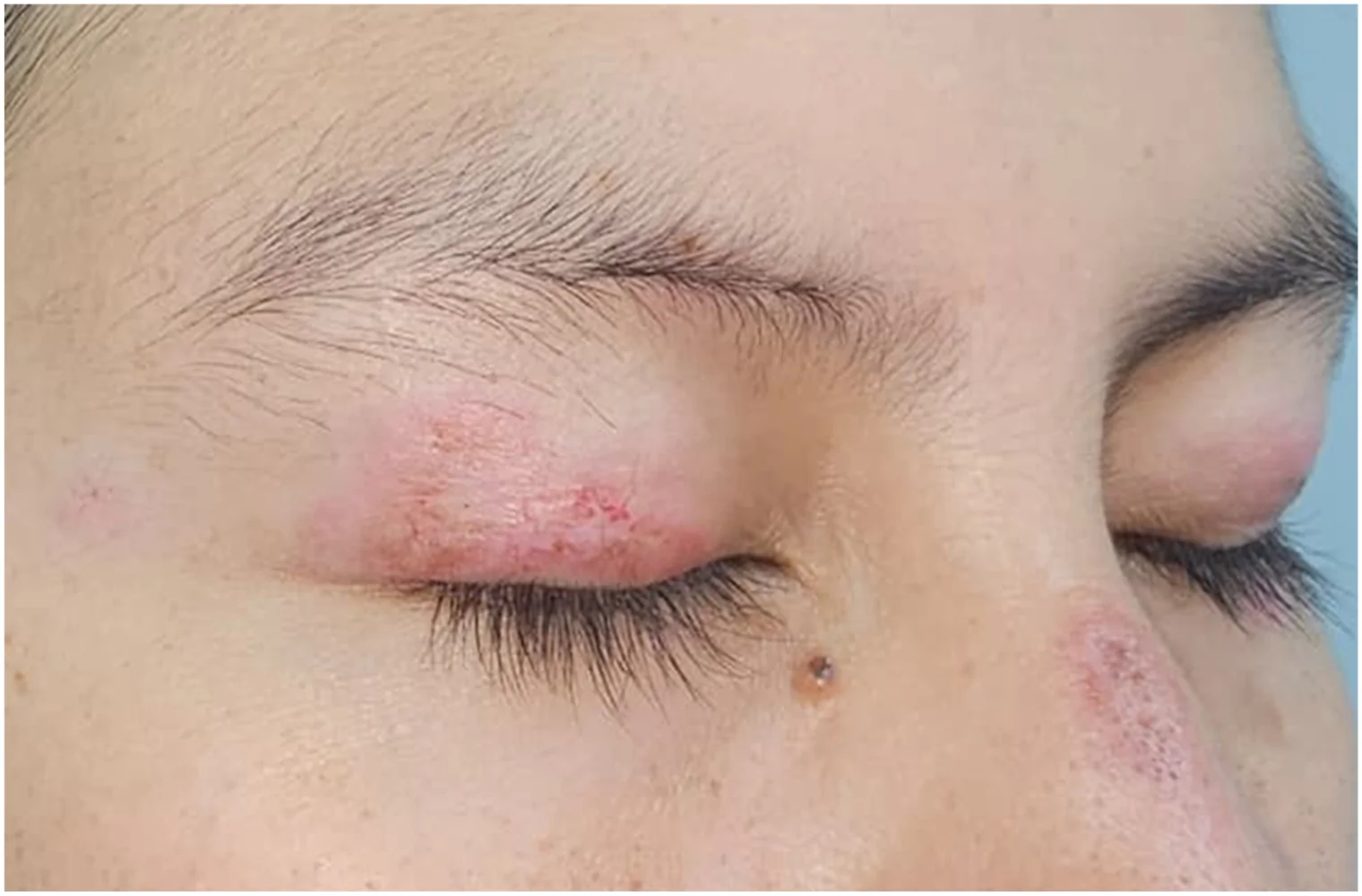
Clinical presentation of discoid lupus with erythematous atrophic plaques on the upper eyelids, periorbital regions, and nasal dorsum for 10 months.

## Dermatoscopic appearance

Linear and tortuous branching vessels, atrophic white areas, and pink structureless area with sparing of the free eyelid margin were observed using a pocket DL200 Hybrid (DermLite) (Fig 2).

**Fig 2:**
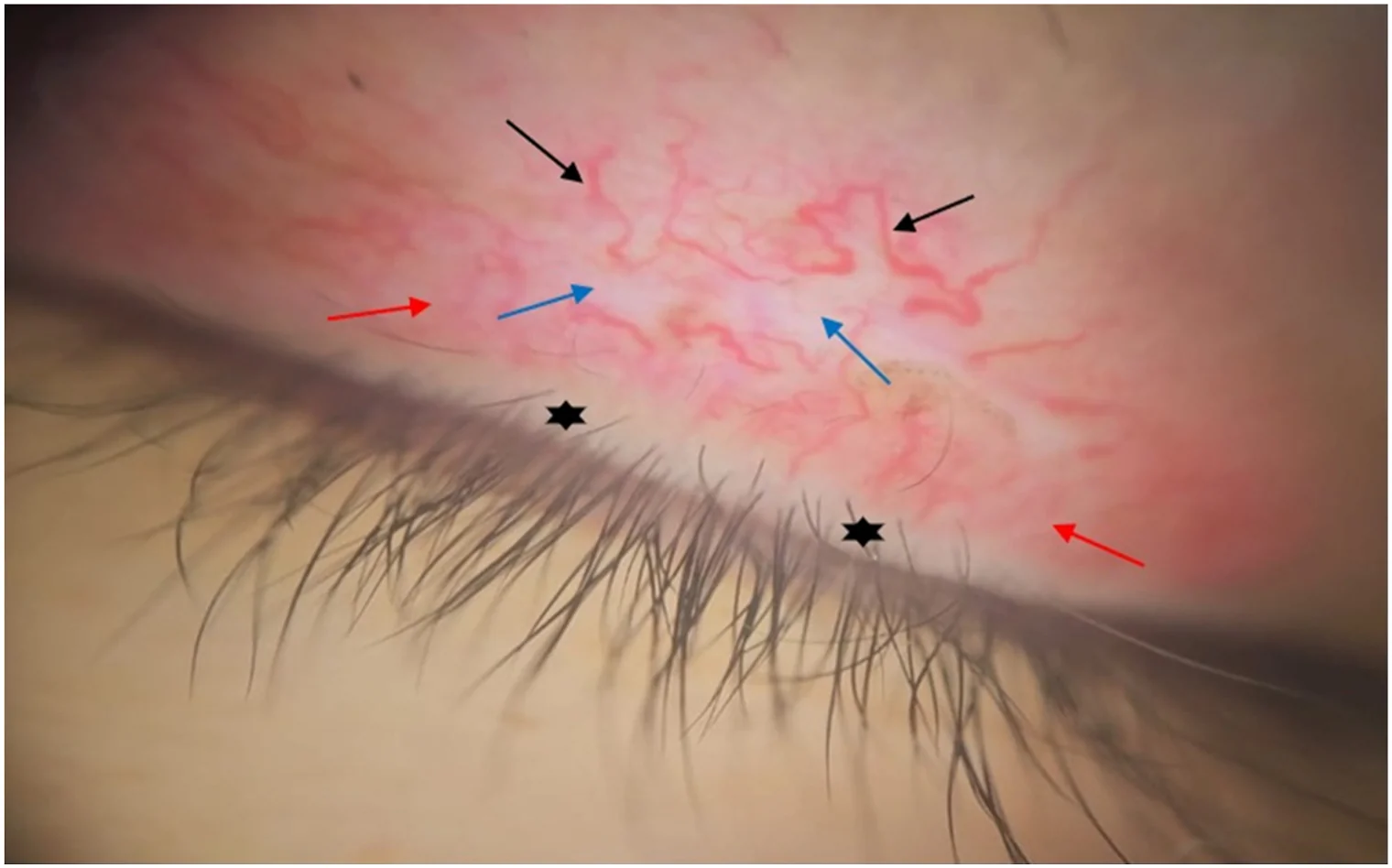
Dermoscopy: linear and tortuous branching vessels (*black arrows*), atrophic white areas (*blue arrows*), and pink structureless area (*red arrows*) with sparing of the free eyelid margin (*asterisk*). (pocket DL200 Hybrid; original magnification: ×20.)

## Histologic diagnosis

The dermatopathology shows vacuolar alteration of the basal layer and dense perivascular and periadnexal inflammatory infiltrate, with interstitial mucin deposition (Fig 3). Discoid lupus erythematosus constitutes the most prevalent subtype of cutaneous lupus erythematousus.^1^

**Fig 3:**
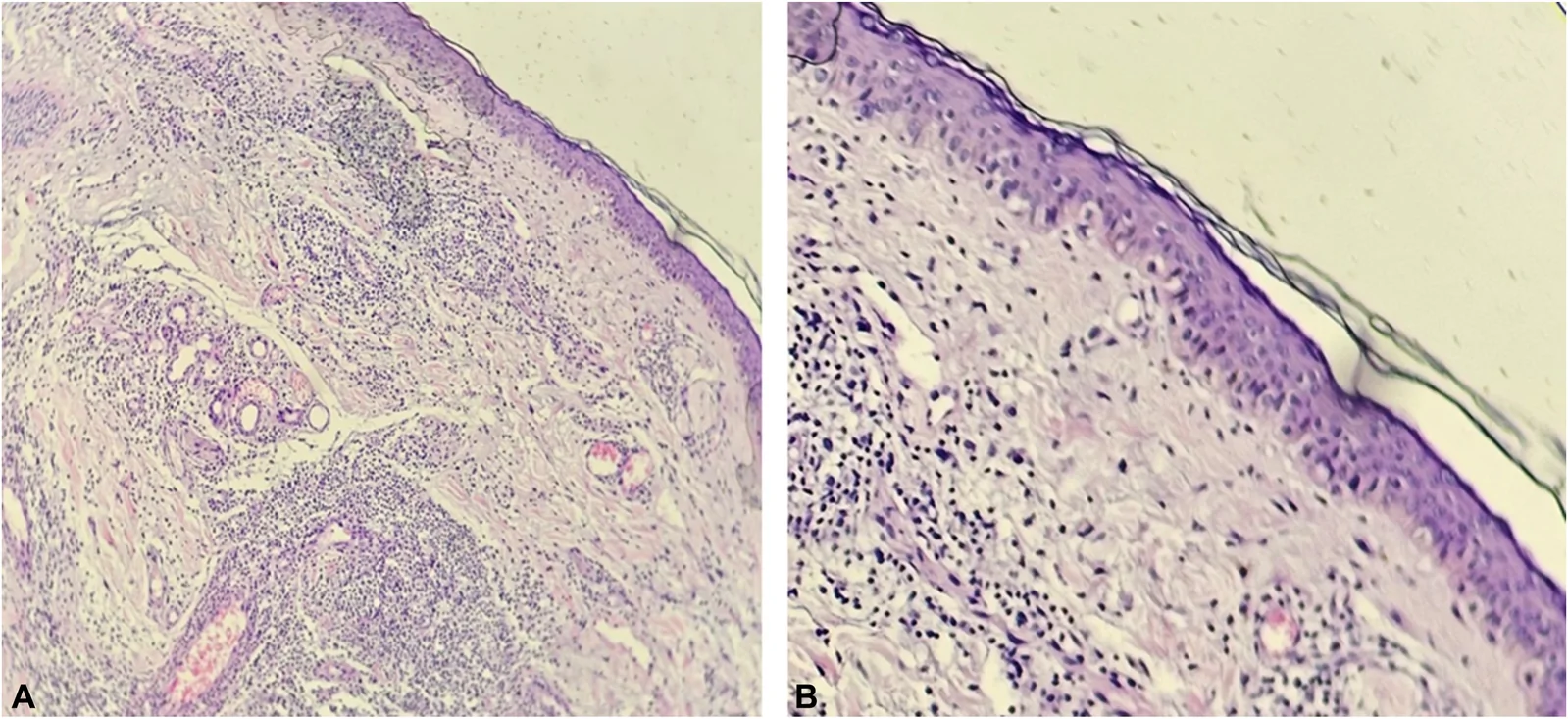
**A,** Hematoxylin-eosin staining of eyelid showing dense perivascular and periadnexal inflammatory infiltrate, with interstitial mucin deposition. **B,** Vacuolar alteration of the basal layer and prominent superficial dermal vessels.

Dermoscopy is valuable for identifying specific features of inflammatory lesions and for aiding in the exclusion of neoplastic processes. Scalp and face are the most frequently effected areas, lesions may also affect the upper trunk, upper extremities, and mucosal surfaces.^2^ Unlike facial discoid lupus erythematosus, in which follicular plugs, perifollicular white halos, and adherent scales are common dermatoscopic findings, eyelid involvement is characterized predominantly by structureless areas, minimal or absent scaling, and telangiectasias, likely reflecting the thin skin of the eyelids. Eyelid involvement is uncommon in discoid lupus erythematosus, occurring in approximately 5% to 6% of patients, and typically presents bilateral.^2^

## Conflicts of interest

None disclosed.
